# Current Progress in Nitrogen Fixing Plants and Microbiome Research

**DOI:** 10.3390/plants9010097

**Published:** 2020-01-13

**Authors:** Kishan Mahmud, Shiva Makaju, Razi Ibrahim, Ali Missaoui

**Affiliations:** 1 Center for Applied Genetic Technologies, University of Georgia, Athens, GA 30602, USA; Kishan.mahmud25@uga.edu; 2 Department of Crop and Soil Sciences, University of Georgia, Athens, GA 30602, USA; shmakaju@uga.edu; 3 Institute of Plant Breeding, Genetics and Genomics, University of Georgia, Athens, GA 30602, USA; Razi.Ibrahim@uga.edu

**Keywords:** biological nitrogen fixation, nitrogenase, *nif* genes, legumes and nodules, associative nitrogen fixation, soil microbiome, rhizosphere

## Abstract

In agroecosystems, nitrogen is one of the major nutrients limiting plant growth. To meet the increased nitrogen demand in agriculture, synthetic fertilizers have been used extensively in the latter part of the twentieth century, which have led to environmental challenges such as nitrate pollution. Biological nitrogen fixation (BNF) in plants is an essential mechanism for sustainable agricultural production and healthy ecosystem functioning. BNF by legumes and associative, endosymbiotic, and endophytic nitrogen fixation in non-legumes play major roles in reducing the use of synthetic nitrogen fertilizer in agriculture, increased plant nutrient content, and soil health reclamation. This review discusses the process of nitrogen-fixation in plants, nodule formation, the genes involved in plant-rhizobia interaction, and nitrogen-fixing legume and non-legume plants. This review also elaborates on current research efforts involved in transferring nitrogen-fixing mechanisms from legumes to non-legumes, especially to economically important crops such as rice, maize, and wheat at the molecular level and relevant other techniques involving the manipulation of soil microbiome for plant benefits in the non-legume root environment.

## 1. Introduction

A healthy, functioning soil ensures nutrient cycling for optimum plant growth for agricultural production [1]. However, agricultural productivity is often limited by available soil nutrients, especially nitrogen [2]. Nitrogen is not present in soil parent material despite the fact that nitrogen content in the atmosphere is highest among all the atmospheric gases [3]. Hence, soil nitrogen input for plant nutrition and crop productivity largely depends on organic matter degradation, synthetic fertilizer applications, and biological nitrogen fixation (BNF) via nitrogenase enzyme activity [4,5]. This limited bio-availability of N and the escalating reliance of crop growth on N have created a colossal N-based fertilizer industry worldwide [6,7]. Nitrogenous fertilizer production currently represents a significant expense for the efficient growth of various crops in the developed world. Synthetic N fertilizers are currently used in grain, grass, and fruit productions (about 60% for cereals and 10% with irrigated rice production) [8]. More than 50% of the applied N-based fertilizer is used by the plants and the remaining can be subjected to losses like surface runoff and leaching leading to nitrate contamination of soils and groundwater. In terms of energy efficiency, moreover, manufacturing nitrogen-based fertilizers requires six times more energy than that needed to produce either phosphorous or potassium based fertilizers [9]. Therefore, reducing dependence on nitrogenous fertilizers in agriculture in the developed world and developing countries may lead to potential gains in an agricultural setting. Biological nitrogen fixation (BNF) in economically important food and forage crops [10] has drawn attention to achieve sustainable agricultural goals in both both hemisphere of the world [11]. In livestock production systems in southeastern USA, strategically planting nitrogen-fixing legumes in cattle pastures has shown to increase the available soil nitrogen [12], thereby reducing the need to apply synthetic nitrogen sources. The diazotrophic microorganisms from bacteria or archaea domains are responsible for BNF and only some prokaryotes are able to use atmospheric nitrogen through BNF by encoding nitrogenase, an enzyme that catalyzes the conversion of N_2_ gas to ammonia (NH_3_) [8,13,14]. Despite the phylogenic and ecological diversity among diazotrophic bacteria and their hosts, a synchronized interaction is always a prerequisite between the microbial entities and the host plant to achieve a successful nitrogen fixation system. The importance of this process is enormous as it reduces the dependence on nitrogen fertilizers for plants and thus, for agriculture overall. It has been estimated that worldwide, biological nitrogen fixation produces roughly 200 million tons of nitrogen annually [15,16]. In fact, nearly 50% of the total nitrogen in crop fields is the contribution of BNF by diazotrophic bacteria of the total biosphere nitrogen [17]. Moreover, fixed nitrogen can also be transferred to intercropped non-legumes in the case of mixed cropping systems, such as the soybean–wheat system, or the next season crops in crop rotation [18]. In this review article, we explore current developments concerning the limitations and potential promises of nitrogen fixation in legumes and non-legumes.

## 2. Biological Nitrogen Fixation (BNF)

Nitrogen fixation is a dynamic and high energy demanding process [19]. The pathway for the biological reduction of inert N_2_ into the reactive compound NH_3_ (ammonia) under micro-aerobic conditions is as follows:

N_2_ + 8H^+^ + 8e^−^ + 16Mg-ATP → 2NH_3_ + H_2_ + 16Mg-ADP + 16 P (1)

Free-living diazotrophs correspond to a small fraction of the plant rhizospheres ecosystem, and they belong to alphaproteobacteria (*Rhizobia*, *Bradyrhizobia*, *Rhodobacteria*), betaproteobacteria (*Burkholderia*, *Nitrosospira*), gammaproteobacteria (*Pseudomonas*, *Xanthomonus*), firmicutes, and cyanobacteria [20]. However, their presence, function, and importance can be explained by the “black queen” hypothesis which predicts that in free-living microbial communities, only a few “helpers” that carry the heaviest weight in terms of functions, such as high energy-requiring nitrogen fixation, support the rest of the flora and fauna population or the “beneficiaries” that rely on the “helpers” or the “beneficials” for nitrogen needs [21].

The symbiotic relationship between soil bacteria, collectively known as rhizobia (which includes the genera *Rhizobium*, *Bradyrhizobium*, *Mesorhizobium*, and *Sinorhizobium*), and legume roots generates nodules (a new differentiated special organ) that fix atmospheric nitrogen through the action of the nitrogenase enzyme [22]. BNF by plants and its bacterial associations represent an important natural system for capturing atmospheric N and processing it into a reactive form of nitrogen through enzymatic reduction. BNF is considered an extremely sensitive process influenced by nutrient and environmental conditions and enables a plant to supply all or part of its requirements through interactions with endo-symbiotic, associative, and endophytic symbionts, thus offering a competitive advantage over any non-nitrogen-fixing plants [15,23,24,25,26]. The highly conserved nitrogenase complex in free-living and symbiotic diazotrophs enables them to participate in various types of associations/interactions with their host plants. BNF by plant–rhizobia symbiotic systems is mediated by endosymbiotic interaction when plants develop root nodules; in legumes and rhizobia, gram-negative alpha proteobacteria are the most common microbial species that associate (endo-symbiotic interaction) with legumes of the Fabaceae (Papilionaceae) family [27,28,29]. Actinomycetes such as the *Parasponia* species (family Cannabaceae) and *Frankia* sp. that associate with a broad spectrum of actinorhizal plants are well documented in nitrogen fixation as well [8]. Cyanobacteria (mainly *Nostoc* sp.) have also been found to colonize different plant organs, either intracellularly in the family Gunneraceae or extracellularly in *Azolla*, *Cycadaceae*, liverworts and hornworts. Associative nitrogen fixation (ANF) and/or endophytic symbioses are often observed among diazotrophs, such as *Azospirillum* spp., *Azoarcus* spp. and *Herbaspirillum*, with a wide variety of plant roots including cereals. The nitrogenase protein, as well as the associated proteins and non-proteins forming nitrogenase enzyme, are sensitive to the presence of oxygen [30]. For this extreme sensitivity to oxygen, obligate anaerobes such as *Clostridium pasteurianum* are ideal candidates for nitrogen fixation; however, facultative anaerobes such as *Klebsiella oxytoca* are also capable of fixing nitrogen but only when the oxygen is absent in the system [31]. Obligate aerobes, such as *Azotobacter vinelandii* can also shield nitrogenase from oxygen and perform nitrogen fixation by consuming oxygen via cytochrome oxidases [31,32].

### 2.1. The Nitrogenase Protein and Nodule Formation

As mentioned earlier, a protein complex called nitrogenase (composed of enzymes with metal co-factors) makes nitrogen fixation possible in plants. The first one is dinitrogenase and the second one is dinitrogenase reductase [33]. According to the active site co-factor binding metal, there exist three types of dinitrogenase in nature. (a) Molybdenum (Mo) nitrogenase; it is most abundant and carries the most significance in the nitrogen-fixing bacterial and archaeal niche and the alternative vanadium (V) and iron-only (Fe) nitrogenases [34]. The molybdenum dependent dinitrogenase is formed by *nifD* and *nifK* gene products and dinitrogenase reductase is a homodimer of the *nifH* gene product [30,35]. It is well documented that molybdenum nitrogenase is produced in all diazotrophs in nature, while some produce the vanadium or iron nitrogenase addition to Mo-nitrogenase [36,37]. The rhizobium bacteria residing in nodules fix atmospheric nitrogen gas to NH_3_, which plants can assimilate via glutamine synthase to form glutamine. In response, the bacteria derive plant carbohydrates, mainly as malate for food and an energy source for nitrogen fixation. Nodules are very complex structures, containing several processes which operate and interact at distinct levels. The process of nodule formation requires a coordinated exchange of signals between the two symbiotic partners [38]. Bacteria had their symbiotic genes first characterized by transposon mutagenesis; this achieved the definition of over 50 nodulation genes (Nod and Nol) in bacteria, and about the same number controlling nitrogen fixation; thus many nod- and fix-bacterial strains exist in many species of rhizobia. Legume–rhizobium symbiosis starts with molecular signaling between the two partners. Early nodulation gene cascades in legumes. Plants release signals such as flavonoids (e.g., the flavone 7,4 dihydroxyflavone and the isoflavone genistein) which are picked up by compatible bacteria in the rhizosphere [39,40] leading to the production of Nod factors (NF) which trigger early events in the nodulation process [41,42]. This triggers the downstream gene cascade including those involved in nucleoporin, cation channels, calcium spiking, early nodule expression, and cytokine signaling leading to cortical and pericyclic cell divisions, and concomitant bacterial infection. Rhizobia are entrapped by root hair curling after the Nod factor has been perceived, which results in initiating the formation of infection thread (a tubular structure). This infection thread facilitates the penetration of root hair cells and adjacent cortical cells [43]. Cell divisions in cortical and pericycle occur simultaneously resulting in the formation of the nodule primordium. Bacterial cell division facilitates the rhizobial traveling through the infection thread and is eventually freed into the induced nodule primordium cells [44,45]. As nodules mature with time, bacteria are enclosed within the symbiosome membrane, resultant from an inverted plasma membrane of plant origin. In this encapsulated chamber, the bacteria experience a micro-aerobic environment (lower oxygen concentration) and differentiate into bacteroids, fixing diffused nitrogen gas using their nitrogenase enzyme complex [46,47]. Depending on whether or not the meristem remains active for the life of the nodule, two main types of nodules are formed on the various legume species, (i) indeterminate or (ii) determinate. In the case of determinate nodules, nodular meristematic activity is terminated early and is usually initiated sub-epidermally in the outer cortex, thus giving rise to spherical nodules [48]. In indeterminate nodules, the inner cortex undergoes cell division (anticlinally) followed by periclinal divisions in the pericycle. Here, cylindrical nodules are formed due to more persistent meristems [49,50].

### 2.2. Genes Encoding Nitrogenase Enzyme

The understanding of the genetic basis of this relationship is of paramount importance and essential for the optimization of nitrogen acquisition rates in legumes themselves. Bacterial *nif* genes are well known to encode the components of the nitrogenase enzyme complex. *nifH*, *nifD*, and *nifK* genes encode the structural subunit of di-nitrogenase reductase and the 2 subunits of di-nitrogenase, respectively. Many rhizobial genes have been fully sequenced, for instance, *Mesorhizobium loti*, *Sinorhizobium meliloti*, and *Bradyrhizobium japonicum* [51,52,53]. These proteins have similar sequences and common structures and functions in many diazotrophs, for instance, *Azotobacter vinelandii*, *Herbaspirillum seropedicae*, *Pseudomonas stutzeri*, and *Bradyrhizobium japonicum* [54,55,56,57]. Furthermore, genetic and biochemical analyses revealed that many additional *nif* genes, including *nifE*, *nifN*, *nifX*, *nifQ*, *nif W*, *nifV*, *nifA*, *nifB*, *nifZ*, and *nifS*, play roles in the regulation of *nif* genes and maturation processes of electron transport and FeMo-cofactor biosynthesis and assembly [58,59]. In addition, the *fixABCX* genes first identified in *Rhizobium meliloti* [60,61] and subsequently in other diazotrophs were reported to encode a membrane complex participating in electron transfer to nitrogenase [62]. The degree of specificity between legumes and rhizobia varies. The Nod factors produced by *Rhizobium etli* and *Rhizobium loti* produce identical Nod factors; however, they have distinct host ranges (*Phaseolus* spp. and *Lotus* spp., respectively) [63]. Moreover, different rhizobia nodulating the same plant may excrete completely different Nod factors. For instance, *Rhizobium tropici* and *R. etli* produce different Nod factors (sulfated and acetylfucosylated, respectively), but both are known to nodulate *Proteus vulgaris* [64]. More examples include *Bradyrhizobium elkanii* and *Bradyrhizobium japonicum*, which have a number of mutual hosts, but their Nod factors differ considerably [65].

### 2.3. Marker-Assisted Selection of Biological Nitrogen-Fixing Plants

Several studies have identified QTL associated with traits related to biological N fixation (Table 1 [66,67,68,69]). The QTL markers can be used in marker-assisted selection for breeding plants with better nitrogen fixation attributes. A QTL for the total ureides (acyl derivatives of urea) was identified on chromosome 17 in soybean which explained 13.26% phenotypic variation [70]. Li and the team [71] cloned a candidate gene associated with a major QTL in soybean for increasing nodule size and named it INCREASING NODULE SIZE1 (GmINS1). The overexpression of GmINS1 increased the N content and the biomass of the soybean plant due to an increase in number, biomass, the abundance of infection cells, and nitrogenase activity of large nodules [71]. The result was the opposite when GmINS1 was suppressed by RNA interference [71].

## 3. Host Plant

Plants are associated with a complex microbiome that contributes to plant nutrient assimilation, growth, and defense. Nitrogen-fixing microbial associations are efficient and well-characterized in legumes but are limited in cereals, including maize. Plants contribute substantially toward the organic carbon pool of soil in the form of lysed cells, mucilage, and root exudates [72]. Root exudates are a complex mixture of simple and complex sugars, amino and organic acids, fatty acids and vitamins [73]. Depending on the plant genotype and growth stage, soil texture, nutrient status of soil, water holding capacity of the soil and, most importantly, the rhizosphere microbial communities, the amount and types of exudates will vary. In turn, root exudate composition in the rhizosphere can influence the soil microbial community and availability of macro and micronutrients, especially nitrogen and phosphorus [74]. The root exudate composition also serves as a recruiting complex of unique prokaryotic and eukaryotic populations [75]. More interestingly, the build of specific microbiota by secreting particular carbon sources is often observed, for instance, dicarboxylates in tomato root exudates favor the growth of pseudomonas biocontrol strains and pea plants excrete homoserine to select *Rhizobium leguminosarum* [76,77,78,79,80]. Plants can also defend themselves through the secretion of phytochemicals that can inhibit the growth of certain microbial entities [80]. The ability to tolerate these chemicals can play an important role in the ability to colonize the plant. For example, the PGPR *Pseudomonas putida* is both tolerant of and attracted by the main antimicrobial benzoxazinoid produced by maize (a non-legume plant) [81]. In addition, transgenic plants expressing opine biosynthesis genes shown to redesign current rhizosphere populations to increase the densities of opine-catabolizing bacteria compared to wild-type [82,83].

### 3.1. Symbiotic Nitrogen Fixation in Legume Nodules

In both natural and cultivated ecosystems, legumes supply a significant amount of nitrogen [84,85,86], and the nitrogen fixed by perennial forage legumes can be as high as the amounts of nitrogen fertilizers used in conventional farming practices [87,88]. Moreover, rhizodeposition from legumes is another substantial source of available nitrogen and other essential plant nutrients in rhizosphere [89,90,91]. In temperate forests, a 28% increase in nitrogen availability was reported due to the direct effect of BNF and the indirect effect of rhizodeposition [92]. In North America, several leguminous plants such as pea (*Pisum sativum* L.), faba bean (*Vicia faba* ssp *minor* L.), and dry bean (*Phaseolus vulgaris* L.) are mainly produced for animal and poultry feed [18]. The most common legumes for human consumption are dry bean, chickpea (*Cicer arietinum* L.) and cowpea (*Vigna unguiculata* L.), lentil (*Lens esculenta* L.), pigeon pea (*Cajanus cajan* L.), and peanut (*Arachis hypogea* L.). All these legumes are capable of nitrogen-fixation and are often grown in intercropping or for crop rotation. Nitrogen fixed by symbiotic association of soybean root system with soil bacteria (*Rhizobia*) has a significant contribution to the growth, development, and maturity stages. The increase in nitrogen fixation capacity can be translated to the increase in plant parts including soybean pods. In the field-grown soybean [*Glycine max* (L.) Merrill] cv. Chippewa in a Typic Eutrocrepts soil at physiological maturity (R7), the amounts of nitrogen derived from fixation (*Ndfs*), nitrogen from soil, and ^15^N-labelled fertilizer (*Ndff*) were 47%, 50%, and 3%, respectively [93]. The contribution of nitrogen in soybean pods and seeds was higher from fixed nitrogen (55%) compared to the nitrogen from soil (43%) [93]. Muñoz, Qi [70] observed cultivated soybeans were more efficient in BNF compared to the wild soybeans.

Forage legumes are grown under a broad climatic spectrum, and they have the potential to give higher yield and provide essential nitrogen to the soil. The four major forage legumes, alfalfa (*Medicago sativa* L.), red clover (*Trifolium pratense* L.), subterranean clover (*T. subterraneum* L.), and white clover (*T. repens* L.) together comprise most of the hot and arid regions on earth’s grasslands [94]. Alfalfa (*Medicago saliva* L.) is a cool-season perennial forage legume that obtains nitrogen from the soil and the BNF through symbiotic association of its root nodules with soil bacteria. For its ability to provide fixed nitrogen, alfalfa is increasingly gaining popularity as a companion forage in grass pastures [95]. The fixed nitrogen by alfalfa is not only used by itself but also is transferred to the subsequent crops, which is also termed as a “niche complementarity effect” [96]. For example, the transfer of nitrogen fixed by alfalfa to different grass species such as timothy (*Phleum pratense* L.) and bromegrass (*Bromus inermis* Leyss) was demonstrated using the ^15^N dilution technique [97]. Heichel, Barnes [97] observed the contribution of N transfer from alfalfa to associated grasses in terms of absolute amounts at 5, 20, and 19 kg N ha^−1^ for the three test years, respectively. The direct excretion of N compounds from the root system and the decomposition of root and nodule debris were attributed to this contribution [97].

In addition to economically important crops and forages, considerable attention has been given to several plant species that can produce biofuel while fixing nitrogen. One important example is *Pongamia pinnata*, which in addition to being a medicinal and green manure plant, can nodulate with several strains of both *Bradyrhizobium* and *Rhizobium*; however, best-selected inocula were *B. japonicum* strains CB1809 and USDA110 [98,99,100]. *Pongamia* resembles the general properties seen in annual legumes such as soybean and its nodules actively fix nitrogen where acetylene is reduced by bacterially encoded nitrogenase [101]. However, in addition, many current plant biofuel feedstocks such as oil palm, canola, willow, corn (*Zea mays*), sugarcane, jatropha, sorghum, and even algae may produce abundant fuels but are not nitrogen-fixing species [38].

### 3.2. Nitrogen Fixation in Non-Legumes

In a non-symbiotic system such as rhizosphere-associative nitrogen fixation, nitrogen-fixing bacteria fix the nitrogen by using carbon and energy sources supplied from the environment, and the bacteria release fixed N probably after lysis of the bacterial cells [102,103]. Symbiotic bacteria such as rhizobia and Frankia are located in nodules, whereas in rhizosphere-associative systems, the diazotrophic bacteria are essential in the free-living state and fix nitrogen using the supply of carbohydrates from the environment [104,105] in accord with the excretion of carbohydrates from the roots and the degradation of soil organic matter. In contrast to the legume-rhizobia symbiotic system in nodules, the associations of plants and microbes in rhizosphere soils and plant root interiors form adjusted or adapted nitrogen-fixing systems under physiologically nitrogen-deficient but energy-sufficient conditions. Over the last 50 years, nitrogen fixation in no-leguminous crops and bacterial associations have been investigated elaborately for their agronomic significance. For example, associative nitrogen fixation in sugarcane (Saccharum spp.), sweet potato (*Ipomoea batatas* L.), and paddy rice (*Oryza sativa* L.) are agronomically significant. Active expressions of the di-nitrogenase reductase-encoded gene (*nifH*) phylogenetically similar to those of *Bradyrhizobium* spp. and *Azorhizobium* sp. were abundantly found in the nitrogen-fixing sugarcane stems, sweet potato stems, and storage tubers. *Setaria viridis*, as well as *Setaria italica* (foxtail millet), is capable of securing a significant amount of fixed nitrogen from associations with *Azospirillum brasilense* [106,107]. Other promising associations include *Azoarcus* sp. strain BH72 and Kallar grass and Klebsiella pneumoniae and wheat [108]. A rhizosphere-associated nitrogen fixation can occur in three ways. First, rhizobia employ “crack entry” (a lack of Nod ABC genes, which results in a Nod factor-independent infection process [52,109,110] and invades xylem parenchyma tissues via cortical cells [111] in cut sugarcane stems [112] and sweet potato tuber [113]). Second, under low-oxygen level or micro-aerobic conditions, rhizobia may show free-living nitrogen fixation; for instance, *Bradyrhizobium* spp., nodulates *Aeschynomene* and *Parasponia*, *Azorhizobium caulinodans*, nodulates *Sesbania rostrate*, and *Burkholderia*, nodulates *Mimosa*, and all these rhizobia are capable of fixing nitrogen without a host plant under low-oxygen conditions [114,115,116,117,118,119]. Third, hormones of rhizobia origin that promote the growth of the host plant in accordance with fixed N acquisition; for instance, endophytic rhizobia promoting plant growth [64].

#### 3.2.1. Bacterial Nitrogen Fixation in Sugarcane

*Beijerinckia* sp. from the rhizosphere of sugarcane was first isolated and observed in EMBRAPA Agrobiologia, Brazil [120]. The expression of nitrogenase *nifH* genes was examined by growing sugarcane cut-stems in Japan soils for 50 and 100 days by means of reverse transcription-polymerase chain reaction (RT-PCR) and the sequencing of *nifH* (encoding nitrogenase iron protein) nucleotides [112]. In that study, *nifH* sequences showed similarities with *Bradyrhizobium* spp. and *Azorhizobium caulinodans*, which suggested that the propagation of these *nifH* carrying *Bradyrhizobium* spp. may be a key factor in the endophytic nitrogen-fixation in the free-living state.

#### 3.2.2. Bacterial Nitrogen Fixation in Sweet Potato

In growing sweet potato (*Ipomoea batatas* L.), farmers use relatively infertile soil and apply a small dosage of chemical fertilizers [121,122]. *Azospirillum* sp. was first identified in fibrous roots and storage root peels of sweet potato, and in the same study, additional investigations also indicated a high input of total nitrogen in sweet potato stems and tubers by endophytic *Bradyrhizobium* spp. [121]. In recent years, an isolated endophytic diazotroph, *Bradyrhizobium* sp. strain AT1 [113] showed a *nifH* sequence similarity to Aeschynomene stem-nodulating *Bradyrhizobium* sp. ORS391 [123].

#### 3.2.3. Bacterial Nitrogen Fixation in Paddy Field

Much higher nitrogenase activities in a paddy rice-soil system were detected compared to the paddy soil without rice plants and in an upland rice–soil system [124]. In flooded soil, the root–soil interface has been proposed as the nitrogen-fixing site and the bacteria sustaining such nitrogen fixation activity under dark in flooded conditions were thought to be heterotrophic diazotrophs such as *Azotobacter* and Clostridia [124,125]. Later, in long term repeated pot experiments at the IRRI (International Rice Research Institute) [126], nitrogen fixation by not only photosynthetic cyanobacteria but also consistently by the heterotrophic diazotrophs utilizing root secretions of carbonaceous origin in the rhizosphere was observed [127]. In addition, a positive nitrogen balance was calculated, suggesting significant atmospheric nitrogen input in paddy rice fields [128]. From *nifD* (a nitrogenase protein-encoded gene) segments from crude root DNA, cloned *nifD* genes similar to those of γ-proteobacteria (*Azotobacter vinelandii*) and α-proteobacteria (*Bradyrhizobium japonicum*) were detected [129,130].

#### 3.2.4. Maize Mucilage and Microbiota Association for Nitrogen Fixation

A recent study carried out in nitrogen-depleted fields of Oaxaca, Mexico, demonstrated that the mucilage associated with the aerial roots of Sierra Mixe maize can aide a complex diazotrophic microbiome that can encode active nitrogenase, and the fixed nitrogen (29% to 82% of the plant nitrogen was derived from atmospheric nitrogen) can efficiently travel from the nitrogen-fixing microbiota to host plants [131]. In maize, aerial roots are known for enhancing nutrient and water uptake as well as an efficient gaseous exchange between plant tissue and the atmosphere [132,133,134].

#### 3.2.5. Bacterial Nitrogen Fixation in Switchgrass

Switchgrass (*Panicum virgatum* L.) is a warm-season C4 grass. It is native to the tallgrass prairies of North America, and it has been well-studied for its use as a forage grass and more recently for its potential as a cellulosic biofuel [135,136]. In the absence of substantial nitrogen deposition or soil organic nitrogen unavailability, rhizobia-associated nitrogen-fixing is solely responsible for nitrogen supply and switchgrass can incorporate recently fixed nitrogen into its tissues [137,138] and diverse communities of nitrogen-fixing bacteria are present in switchgrass rhizospheres such as *Burkholderia* spp., and *Ralstonia taiwanensis* of beta-proteobacteria have been found in the tissues of switchgrass which are known to form root nodules on host plants of *Mimosa* [139].

## 4. Current Strategies and Tools for Engineering Symbiotic Nitrogen Fixation in Non-Legumes

Although symbiotic nitrogen fixation is largely limited to legumes, there is an array of microorganisms, including some diazotrophs that inhabit the rhizosphere of other crop plants, which have been shown to enhance plant growth. The mechanisms involved in plants and microbes that lead to the formation and function of symbioses will help us in transferring these traits and processes to non-leguminous crops, especially in cereals. Advanced understanding of BNF, bacterial association with non-leguminous plants, and the microbial community composition of the rhizosphere population have led to several future research ideas for scientists; for instance, engineering non legume plants to nodulate and establish symbiotic nitrogen fixation, and the formulation of new associations between nitrogen-fixing microorganisms and crop plants [140,141]. Studies of evolutionary genomics suggest that relatively few genetic elements are needed to bestow nitrogen-fixation capabilities from legume to non-legume plants [142]. Transferring nitrogenase to plants requires the concatemerization of bacterial genetic units to create a minimum set of three genes [143]. The introduction of nitrogenase-encoding bacterial *nif* genes into non-legumes is challenging due to the complex nature of nitrogenase biosynthesis and the extreme sensitivity of nitrogenase to the presence of oxygen. Extensive genetic and biochemical studies have identified the common core set of genes/gene products required for functional nitrogenase biosynthesis [144].

In addition, potential subcellular (micro-pockets of air) low-oxygen environments offered via plastids and mitochondria to express active nitrogenase in plants making this engineering strategy feasible [145]. Although the nitrogen-fixing symbiosis is restricted to legumes, several components of the legume symbiotic signaling (SYM) pathway also play a role in the arbuscular mycorrhizal symbiosis. Many plants, including cereals, can form arbuscular mycorrhizal associations but lack the ability to form root nodules that can fix nitrogen. Like in legumes, the legume symbiotic signaling pathway (or SYM) also promotes the arbuscular mycorrhizal symbiosis. Since cereals contain the SYM pathway for arbuscular mycorrhizal associations, thus, this association can be engineered to perceive the rhizobial signaling molecules to activate this pathway, as well as by engineering its outputs of activation into an oxygen-limited nodule-like root organ for nitrogen fixation [64]. Recent phylogenomic studies suggest that a small set of genes could convert a species in association with arbuscular mycorrhizal fungi into a nitrogen-fixing symbiont [146,147].

In cereal crops, mitochondria and chloroplasts (“nitroplast”) [38] in plant cells are envisioned as suitable sites for performing the high energy-requiring nitrogenase enzyme production; however, a challenge for this approach would be the oxygen evolved by chloroplasts during photosynthesis that may be detrimental for the formation of the nitrogenase enzyme complex. A possible solution could be the temporal separation of photosynthesis and nitrogen fixation, which means the expression of *nif* gene only to dark periods (nights) or only in root systems (non-photosynthetic parts of plants) [19]. Also, a carbon secretion based approach in which a specialized carbon source encourages enhanced competition for carbon among nitrogen-fixing populations can also be utilized to establish appropriate signals between cereal crops and nitrogen-fixing microbes for effective colonization [64].

Previous studies have reported the influence of novel nutritional resources in the selection of microbial populations in the rhizosphere [83,148]. For instance, pea root mucilage is the sole source of carbon for some *Rhizobium* sp., *Burkholderia* sp., and *Pseudomonas* sp. [149]. Pursuing this “biased rhizosphere” approach to favor the growth of an introduced diazotroph which is able to use the novel rhizodeposition will involve the identification of appropriate plant and bacterial signals, receptors, and target genes [150]. Although transferring nitrogen fixation traits to crops beyond legumes has complex engineering problems, especially in the case of cereals, however, they might restructure the way cereal crops are grown. Even a small increase in available nitrogen in these self-supported nitrogen-fixing cereals will enable a substantial yield increase in the low-input farming systems of developing countries [151]. Moreover, in eukaryotes, some components of nitrogenase enzyme, for instance, active dinitrogenase reducatase can be expressed by mitochondrial targeting in yeast or plastid-targeting strategies in tobacco [69,152,153]. There is even an effort to synthesize an entire eukaryotic genome (Yeast 2.0). This expression of dinitrogenase in yeasts has important implications, especially for efficient nutrient uptake such as phosphate in cereal crop root habitats [131,154].

Opine molecules produced by transgenic plants are known to boost their rhizosphere with opine catabolizing bacteria; however, there is a risk of populating the rhizosphere with chemical compounds originating from pathogenic organisms [82,83,148]. Rhizopines are a rare group of compounds produced by a few species of rhizobia inside legume nodules and are exuded into the rhizosphere; namely, scyllo-inosamine 1 (SIA) and 3-*O*-methyl-scyllo-inosamine 2 (3-*O*-MSI) are believed to be suitable for ideal chemical signaling in the realm of trans-kingdom signaling between plants and rhizosphere bacteria, although engineering rhizopine-producing plants have not seen much success [155,156,157,158]. Rhizopines serves as the energy source (carbon and nitrogen) for Rhizobia and the gene responsible for rhizopine synthesis is (mosABC) and catabolism is (mocCABRDEF), which have been identified in the wild-type rhizobium Sinorhizobium meliloti L5-30 [159,160]. In the continued efforts in rhizosphere engineering of cereal crops, a recent study has successfully transferred the rhizopine biosynthesis genes into Hordeum vulgare (barley) [161]

## 5. Conclusions

Biological nitrogen fixation in plants can be a sustainable source of nitrogen and may divert our current dependence on industrial nitrogen production. This is especially true for food production in the developed world where agricultural production is still based on higher-yielding varieties and hybrids but with a simultaneous increase in inorganic nitrogen application. Not all the applied nitrogen in agricultural production is taken up by plants and the unused nitrogen has negative impacts on the environment, extending from eutrophication in nearby water bodies if the excess nitrogen is washed up by rainfall or surface runoff or nitrate poisoning in livestock. Future research should be focused on the efficient and strategic use of nitrogen-fixing legume plants, the use of higher legume plants such as *Pongamia pinnata*, in planned agroforestry, selecting highly competitive inoculants, and the use of non-legumes for nitrogen fixation. Artificial symbioses, associative nitrogen fixation in non-legume plants, especially in cereals such as rice, wheat, maize, targeted or biased rhizosphere, and understanding of endosymbiotic and endophytic nitrogen fixation with non-legume plants are some of the approaches that should be investigated to a greater extent.

## Figures and Tables

**Table 1:** Major genomic loci detected for BNF in different legume species [66,67,68,69].

Species	Chromosome Number	QTL or Marker Interval	Plant Response	QTL-Effect, R^2^ (%)
Common bean (*Phaseolus vulgaris* L.)	7	*Ndfa7.1^DB,SA^*	N derived from atmosphere (Ndfa)	14.9
Soybean [*Glycine max* (L.) Merr.]	16	*qBNF-16*	Nodule size & number	15.9–59
Soybean [*Glycine max* (L.) Merr.]	17	*qBNF-17*	Nodule size & number	12.6–18.6
*Lotus japonicus*	2	TM0550–TM0324	Acetylene reduction activity per plant (ARA/P)	15.1
*Lotus japonicus*	2	TM0550–TM0002	ARA per nodule number (ARA/NN)	11.1
*Lotus japonicus*	4	TM0664	ARA per nodule weight (ARA/NW)	10.8
*Lotus japonicus*	5	TM1417–TM0095	ARA per nodule weight (ARA/NW)	13
*Lotus japonicus*	3	TM0083	Nodule number (NN)	21.6
*Lotus japonicus*	1	TM0113–TM0805	Stem length (SL)	13.3
*Lotus japonicus*	1	TM0027–TM0063	Shoot length without inoculation (SL bac−)	16.7
*Lotus japonicus*	1	TM0113–TM0805	Shoot length without inoculation (SL bac−)	16
*Lotus japonicus*	5	TM0095–TM0909	Shoot dry weight without inoculation (SW bac−)	10.7
Cowpea [*Vigna unguiculata* (L.) Walp.]	4 (Likage group)	2_12850/2_54418	Nodule number	48.4
Cowpea [*Vigna unguiculata* (L.) Walp.]	6 (Likage group)	2_11936/2_49231	Nodule fresh weight	21.4
